# Intranasal oxytocin: miracle cure after trauma?

**DOI:** 10.3402/ejpt.v6.27631

**Published:** 2015-04-08

**Authors:** Miranda Olff, Mirjam Van Zuiden, Saskia B. J. Koch, Laura Nawijn, Jessie L. Frijling, Dick J. Veltman

**Affiliations:** 1 Department of Psychiatry, Academic Medical Center, University of Amsterdam, Amsterdam, The Netherlands; 2 Arq Psychotrauma Expert Group, Diemen, The Netherlands; 3 Department of Psychiatry, VU University Medical Center, Amsterdam, The Netherlands

***Background***: In popular media and on Internet, the neuropeptide oxytocin is often advertised as a miracle drug that cures all types of disorders, reduces stress, saves marriages, all conveniently with a nasal spray. Here we will present the effects of intranasal oxytocin on brain function in recently traumatized individuals and patients with posttraumatic stress disorder (PTSD) and discuss clinical implications and further research. PTSD is characterized by exaggerated fear responses to threat and trauma-related stimuli, reflected in altered neural salience processing and emotion regulation (Koch et al., 2014). In addition, many PTSD patients report anhedonia, emotional numbing, and social detachment, reflected in decreased reward sensitivity (Nawijn et al., 2015). Interestingly, dysregulations in these domains also appear to be associated with increased PTSD risk upon trauma exposure and treatment non-response. Currently, there still is a high need for development of effective preventive interventions for PTSD, that can be administered early after trauma, as well as a need for novel adjuvant interventions that augment treatment response to evidence-based psychotherapy for PTSD (i.e., medication-enhanced psychotherapy [MEP]) (see also Hendriks & De Kleine, 2015).

In 2011, we received a large grant (ZonMW TOP) to perform a series of functional neuroimaging studies on the effects of a single intranasal administration of the neuropeptide oxytocin on neural emotional and reward processing in PTSD patients versus healthy traumatized controls, and in recently traumatized individuals at high risk for PTSD due to high levels of distress acutely after trauma.

These studies were based on literature showing that intranasal oxytocin impacted a variety of the behavioral, neural, and neuroendocrine dysregulations observed in PTSD patients and individuals vulnerable for PTSD. Thus, intranasal oxytocin appeared to be a promising candidate for PTSD prevention and augmentation of treatment response (Olff, 2012; Olff, Langeland, Witteveen, & Denys, 2010).

***Objective***: We aimed to investigate the neurobiological mechanisms of fear (i.e., salience processing and emotion regulation) and reward processing underlying oxytocin's potential therapeutic effect for PTSD prevention and treatment. Regarding neural salience processing and emotion regulation, it had been found that oxytocin administration in healthy individuals dampened amygdala reactivity toward threat-related stimuli (Kirsch et al., 2005) and toward conditioned stimuli associated with receiving shocks (Petrovic, Kalisch, Singer, & Dolan, 2008). Therefore, we hypothesized that oxytocin administration would dampen exaggerated neural fear of salience processing and would increase connectivity within neural emotion regulation networks in both PTSD patients and in recently traumatized individuals at increased PTSD risk (Frijling et al., 2012; Frijling et al., 2014). Regarding neural reward processing, it appeared that oxytocin administration increased sensitivity for positive social stimuli by stimulating key brain areas of the reward pathway (such as the nucleus accumbens), a system of brain areas important for processing of positive stimuli, in healthy individuals. Therefore, we hypothesized that oxytocin administration would increase neural sensitivity for (social) reward and thereby affect emotional numbing and social detachment in PTSD patients, allowing PTSD patients to benefit more from provided social support, including the support offered by therapists during treatment (Olff et al., 2010).

***Results***: Here we present a summary of the first results of our fMRI studies on the effects of a single oxytocin administration on fear and reward processing in PTSD patients versus healthy traumatized controls and in recently traumatized individuals.

Our results show that oxytocin administration dampened amygdala reactivity toward emotional faces in PTSD patients, but increased amygdala reactivity toward (negative) emotional faces in both recently traumatized individuals with high levels of distress as well as highly traumatized individuals without psychopathology. Additionally, we found opposing effects of oxytocin administration on functional connectivity of the amygdala with brain areas involved in emotion regulation and salience processing between our study populations. In rest, male PTSD patients showed increased connectivity within the emotion regulation network after oxytocin, while female PTSD patients showed decreased connectivity within the salience network. In contrast, oxytocin administration resulted in decreased resting state connectivity within the emotion regulation network and increased connectivity within the salience network in recently traumatized individuals after exposure to trauma-related script-driven imagery. Regarding neural effects on reward sensitivity, oxytocin administration increased striatal responses to both reward and loss anticipation in PTSD patients and highly traumatized controls.

***Conclusions***: Combined, our findings on the effects of oxytocin administration on neural salience processing and emotion regulation indicate that a single oxytocin administration has differential effects in (recently) traumatized individuals and PTSD patients. This is in line with more recent literature on differential effects of oxytocin administration depending on interpersonal differences and (the interpretation of) the salience of contextual cues (Bartz, Zaki, Bolger, & Ochsner, 2011; Olff et al., 2013). The observed effects of oxytocin administration in our recently traumatized population are contrary to our initial hypothesis, suggesting that caution is warranted regarding administration of oxytocin in recently traumatized individuals. However, for various reasons, it may be expected that repeated oxytocin administration has different effects than a single administration (for review, see Macdonald & Feifel, 2013). Our randomized controlled trial on the effects of a 1-week oxytocin treatment regimen on PTSD prevention, which is currently well underway, will shed more light on the long-term clinical effects of oxytocin administration in recently traumatized individuals.

Our findings on the neural effects of a single oxytocin administration in PTSD patients are in line with our hypotheses. Thus, oxytocin is not a miracle panacea, but our results do support the notion that intranasal oxytocin administration is a promising candidate for augmentation of PTSD treatment response. As a next step, the potential of intranasal oxytocin administration as an adjunctive agent during psychotherapy for PTSD, such as before exposure-based therapies, should be further investigated in a clinical setting, preferably in a randomized placebo-controlled trial.

## Grants and disclosures

The study is supported by grants from ZonMw (Top Grant), the Netherlands organization for Health Research and Development (grant no. 40-00812-98-10041) and the Academic Medical Center Research Council (110614).

All authors declare that they have no biomedical financial interests and no potential conflicts of interest.
